# Multiple Introductions of Domestic Cat Feline Leukemia Virus in Endangered Florida Panthers^1^

**DOI:** 10.3201/eid2501.181347

**Published:** 2019-01

**Authors:** Elliott S. Chiu, Simona Kraberger, Mark Cunningham, Lara Cusack, Melody Roelke, Sue VandeWoude

**Affiliations:** Colorado State University, Fort Collins, Colorado, USA (E.S. Chiu, S. Kraberger, S. VandeWoude);; Florida Fish and Wildlife Conservation Commission, Gainesville, Florida, USA (M. Cunningham, L. Cusack);; National Institutes of Health, Bethesda, Maryland, USA (M. Roelke)

**Keywords:** Florida panther, Puma concolor coryi, endangered species, feline leukemia virus, viruses, domestic cats, FeLV-B, outbreak, zoonoses

## Abstract

The endangered Florida panther (*Puma concolor coryi*) had an outbreak of infection with feline leukemia virus (FeLV) in the early 2000s that resulted in the deaths of 3 animals. A vaccination campaign was instituted during 2003–2007 and no additional cases were recorded until 2010. During 2010–2016, six additional FeLV cases were documented. We characterized FeLV genomes isolated from Florida panthers from both outbreaks and compared them with full-length genomes of FeLVs isolated from contemporary Florida domestic cats. Phylogenetic analyses identified at least 2 circulating FeLV strains in panthers, which represent separate introductions from domestic cats. The original FeLV virus outbreak strain is either still circulating or another domestic cat transmission event has occurred with a closely related variant. We also report a case of a cross-species transmission event of an oncogenic FeLV recombinant (FeLV-B). Evidence of multiple FeLV strains and detection of FeLV-B indicate Florida panthers are at high risk for FeLV infection.

Feline leukemia virus (FeLV) is a common pathogenic infectious disease responsible for high mortality rates for domestic cats, particularly before development of effective vaccines in the 1980s (*1*). Subgroup FeLV-A, which is replication competent and horizontally transmissible, is responsible for most infections (*1*,*2*). Other FeLV subgroups (B, C, D, E, and T) arose after recombination or through mutation (*3*). FeLV causes immunosuppressive, neoplastic, and hematopoietic disorders that correlate with FeLV subgroups (*4*–*6*). Virulent FeLV-B, the most common novel variant, arises after recombination between FeLV-A and endogenous FeLV (EnFeLV) present in the domestic cat genome and resulted in altered cellular tropism (*1*,*7*–*10*). Horizontal transmission of FeLV-B is rare in domestic cats and is believed to require co-transmission with FeLV-A as a helper virus because of its replication-defective nature (*11*,*12*).

FeLV prevalence in domestic cats is variable (prevalence range 3%–18%) (*13*–*16*). FeLV has the capacity to infect nondomestic species including jaguars, bobcats, the critically endangered Iberian lynx, and pumas, most notably the endangered puma subspecies, the Florida panther (*Puma concolor coryi*) (*17*–*21*). In all non-*Felis* spp. FeLV cases, the source was believed to be domestic cats, which serve as the dominant primary host. The genus *Felis* is the only taxon known to harbor enFeLV (*22*). The presence of FeLV genetic sequences in the germline results in recombination between exogenous FeLV and FeLV-A during domestic cat infections and in emergence of more deleterious subgroups (i.e., FeLV-B) that are not considered to be replication-competent in the absence of co-infection with FeLV-A (*12*). It is assumed that felids belonging to genera other than *Felis* are only infected with FeLV-A because they do not harbor enFeLV genomes.

Outbreaks of infection with FeLV have caused concern in endangered felids that have population bottlenecks because the species might be more vulnerable to infection because of reduced genetic diversity. For example, 21% of Iberian lynx sampled during 2003–2007 were FeLV positive; 6 died from FeLV-related disease (*23*). During 2001–2004, an outbreak of infection with FeLV was documented in Florida panthers (*19*). Ten Florida panthers were FeLV PCR positive, and 5 of these panthers were also determined to be antigen ELISA positive. Three deaths were attributed to FeLV-related disease (*19*,*24*). Phylogenetic analysis of a region of the FeLV *env* gene during this outbreak indicated a single circulating FeLV strain, likely following introduction of the virus from a domestic cat (*24*). The Florida Fish and Wildlife Conservation Commission (FFWCC; Tallahassee, FL, USA) attempted to contain the FeLV outbreak by implementing a vaccination campaign spanning 2003–2007 (*19*).

During August 2004–November 2010, ≈125 live-captured or necropsied panthers were tested for FeLV, and no additional cases were detected (FFWCC, unpub. data). However, since December 2010, a total of 6 Florida panthers found dead were positive for FeLV antigenemia. These cases are separated in both time and space from the 2001–2004 outbreak (Figure 1). Four likely scenarios exist to explain the epidemic curve. First, absence of FeLV (2004–2010) might have resulted from complete eradication of the first outbreak virus, followed by introduction of another strain from domestic cats (Figure 2, panel A). Second, contemporary cases might have arisen from infection that persisted but was unrecognized for 6 years (Figure 2, panel B). Third, new cases may have resulted from a combination of scenarios 1 and 2 (Figure 2, panel C). Fourth, new cases might be explained by introduction of multiple strains (Figure 2, panel D).

**Figure 1:**
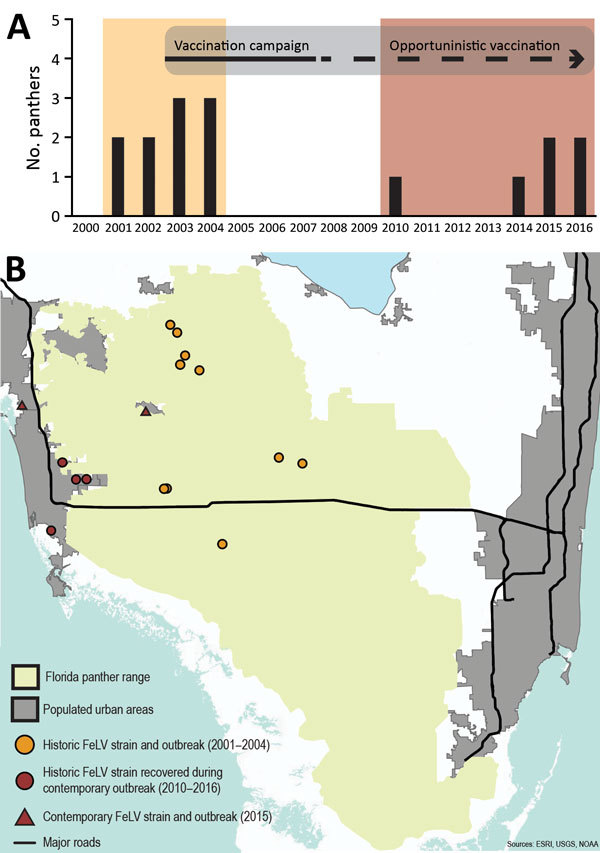
Temporally and spatially distinct FeLV cases in endangered Florida panthers, Florida, USA. A) Incidence of FeLV in live-caught and necropsied Florida panthers. Recaptured panthers are not represented. Different colors indicate first (yellow) and second (red) outbreak events. A vaccination campaign began in 2003 and efforts to actively vaccinate panthers continued until 2007; vaccination has continued opportunistically since the campaign. B) Distribution of historic and contemporary Florida panther FeLV cases in southern Florida. FeLV, feline leukemia virus.

**Figure 2:**
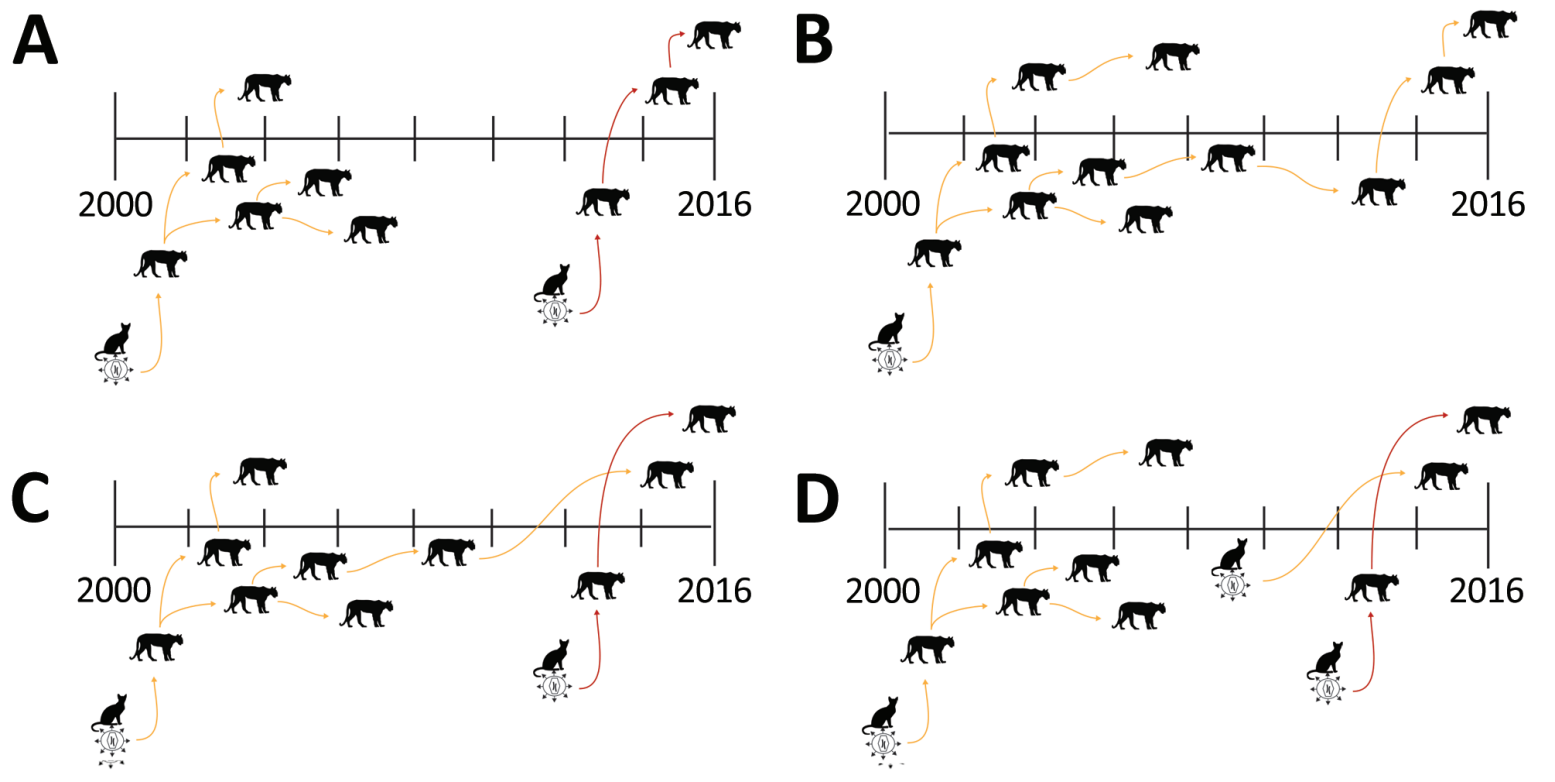
Hypotheses for feline leukemia virus outbreaks in Florida panthers, Florida, USA. A) Secondary infection; B) persistent transmission of 1 virus strain; C) combination of the 2 scenarios; D) multiple infections. Transmission of different virus strains is indicated by orange or red arrows.

In this study, we examined the genetic relatedness between new FeLV isolates and cases described before 2004 with 3 aims. First, we attempted to establish whether recent cases represent a new outbreak or continuation of the prior infection. Second, we sought to determine the genetic relationship between Florida panther and Florida domestic cat FeLV strains. Third, we aimed to gain insights for pathogen–host interactions to better inform management practices and reduce risk for FeLV spillover from domestic cats to endangered felid populations.

## Materials and Methods

### Sample Collection and Processing

FFWCC routinely screens samples collected from Florida panthers for FeLV antigenemia and FIV antibodies by using commercially available test kits (SNAP Combo FeLV/FIV test; IDEXX Laboratories, https://www.idexx.com/en). Surveillance includes animals reported dead or those live captured as part of ongoing health monitoring and population management efforts. We calculated 95% CIs of prevalence for outbreak periods and the intervening quiescent period by using the Wilson score method without continuity correction (*25*).

During 2010–2016, six (2.8%) of the 214 Florida panthers (*P. concolor coryi* UCF149, UCFP 228, FP231, UCFP241, UCFP269, and UCFP275) (Table 1) reported dead to the FFWCC were positive for FeLV antigen during standard postmortem testing on heart, chest, or venous blood collected at necropsy. Lymphoid tissues from FeLV-positive and FeLV-negative controls (i.e., bone marrow, lymph node, spleen, and thymus) in addition to a fibroblast tissue culture from 1 panther (UCFP241R1) were harvested in Florida, stored at −80°C, and shipped to the Feline Retrovirus Research Laboratory at Colorado State University for additional testing.

**Table 1:** Characteristics for Florida panthers tested for FeLV and virus genomes sequenced*

Field sample ID	Laboratory sample ID	Host species	Collection date	State	Full genome	Partial regions (LTR-gag, gag, and env)	GenBank accession nos.
FP115	x1755	*Puma concolor coryi*	2002 Nov 26	FL	No	Yes	MG020270–2
FP122	x1948	*P. concolor coryi*	2004 Jan 30	FL	Yes	Yes	MF681672
FP132	x1955	*P. concolor coryi*	2004 Mar 17	FL	No	Yes (3,688–8,396 nt)	MG020273
UCFP149	x2004	*P. concolor coryi*	2013 Dec 10	FL	Yes	Yes	MF681665
	x2004R1†				Yes	Yes	MF681666
UCFP228	x2271	*P. concolor coryi*	2014 Dec 28	FL	No	Yes	MF681676–678
UCFP231	x2270	*P. concolor coryi*	2015 Jan 20	FL	Yes	Yes	MF681667
UCFP241	x2272	*P. concolor coryi*	2015 Apr 30	FL	Yes	Yes	MF681668 and MF681671 (FeLV-B)
UCFP269	x2274	*P. concolor coryi*	2016 Feb 18	FL	No	Yes	MF681679–682, 2 *env* genotypes from 1 sample
UCFP275	x2273	*P. concolor coryi*	2016 Apr 6	FL	Yes	Yes	MF681669
517278	x1608	*Felis catus*	2011 Nov 10	FL	No	Yes	MF681673–675
517453	x1613	*F. catus*	2011 Nov 10	FL	Yes	Yes	MF681664
BDX387	x2512	*F. catus*	2015 Jul 3	MD	Yes	Yes	MF681670
	x2653	*F. catus*	2018 Jan 6	FL	Yes	Yes	MH116004
	x2655	*F. catus*	2018 Jan 31	FL	Yes	Yes	MH116005

*FeLV, feline leukemia virus; FP, Florida panther; ID, identification; UCFP, uncollared FP. †Recaptured puma.

We isolated genomic DNA from tissues by using bead-beater disruption and phenol-chloroform extraction adapted from Fan and Gulley (*26*). We extracted ≈100 mg of each tissue by using 1.4-mm ceramic spheres in a Fastprep-24 tissue homogenizer (MP Biomedicals Inc., https://www.mpbio.com). Sodium dodecyl sulfate (3 mol/L) was added to the tissue homogenates at a final concentration of 10% and incubated at 37°C overnight. Cell lysates were washed twice with phenol-chloroform. Extracted DNA was concentrated by ethanol precipitation, pelleted, dried, and resuspended in TE buffer.

In addition to tissues from the contemporary outbreak, we analyzed DNA from 3 historically infected (2002–2004; FFWCC) FeLV-infected Florida panthers (FP115, FP122, and FP132), and 4 domestic cats from Florida (x1608, x1613, x2653, and x2655) collected during 2008–2018 for FeLV genomes (Table 1). Florida panther samples were obtained as described (*19*). Domestic cat samples were remnants of archival samples from animals brought to shelters (*27*) or provided by veterinary clinics to FFWCC. An additional FeLV-positive DNA blood sample obtained from a domestic cat (x2512) was included for analysis in this study (Table 1) (*28*).

### FeLV Genome Recovery and Analyses

We sequenced full FeLV proviral genomes (8,448 bp) from 4 domestic cats (1 from Maryland and 3 from Florida), 4 of 6 contemporary Florida panthers (2010–2016), and 1 historic Florida panther (2001–2004) (Table 1). We generated two 5-kb fragments spanning the ≈8.4-kb genome, which were overlapped 1.5 kb. PCRs contained 500 nmol/L of each primer, HiFi Kapa polymerase (Kapa Biosystems, https://www.kapabiosystems.com/region-selector/), 50–200 ng of DNA template, and PCR primers (Table 2) and operated under various cycling conditions. We confirmed FeLV status in our laboratory by using FeLV-PCR and antigen ELISA tests and established protocols (*28*,*29*).

**Table 2:** Primer sequences used for PCR testing of each species and locus for FeLV*

Region	Sequence, 5′ → 3′	Species	Cycling conditions or reference
Full genome (first half)			95°C for 3 min; 30 cycles at 98°C for 20 s; 60°C for 15 s; 72°C for 2 min 40 s; 72°C for 2 min 40 s
Forward	TGAAAGACCCCCTACCCCAAAATTTAGCC	*Puma concolor coryi/Felis catus*	
Reverse	GCGGGTCCATTATCTGAACCCAATACC	*P. concolor coryi/F. catus*	
Full genome (second half)			
Forward	GAGTTCCTTGGAACTGCAGGTTACTGCC	*P. concolor coryi/F. catus*	
Reverse	TGAAAGACCCCTGAACTAGGTCTTCCTCG	*P. concolor coryi*	
Reverse 2	GCTGGCAGTGGCCTTGAAACTTCTG	*F. catus*	
FeLV-B *env*			(*28*)
Forward	CAGATCAGGAACCATTCCCAGG	*P. concolor coryi*	
Reverse	CCTCTAACTTCCTTGTATCTCATGG	*P. concolor coryi*	
LTR-*gag*			
Forward	CGCAACCCTGGAAGACGTTCCA	*P. concolor coryi/F. catus*	95°C for 3 min; 30 cycles at 98°C for 20 s; 60°C for 15 s; 72°C for 15 s; 72°C for 30 s
Reverse	TCGTCTCCGATCAACACCCTGTATTCA	*P. concolor coryi/F. catus*	
*gag*			
Forward	GGACCTTATGGACACCCCGACCAA	*P. concolor coryi/F. catus*	
Reverse	GGAGGGGGTAGGAACGGACGAA	*P. concolor coryi/F. catus*	
*env*			
Forward	CCTTTTACGTCTGCCCAGGGCAT	*P. concolor coryi/F. catus*	
Reverse	TTCCACCAAGCTTCTCCTGTGGTCT	*P. concolor coryi/F. catus*	

*The first half-genome primer set for FeLV sequencing (F/5.2R) spanning 5′-LTR/3′-pol was the same for the FeLV-positive Florida panthers and domestic cats. Reverse half-genome primers were designed to avoid amplification of domestic cat enFeLV and therefore differed for Florida panthers and domestic cats. FeLV, feline leukemia virus.

We extracted PCR products after electrophoresis from a 0.7% agarose gel, purified them by using a MEGAquick-spin Total Fragment DNA Purification Kit (iNtRON Biotechnology, http://jhscience.com/index.php?manufacturers_id=1), and cloned them into a pJET 1.2 blunt vector by using the CloneJET PCR Cloning Kit (Thermo Fisher Scientific, https://www.thermofisher.com/us/en/home.html). We transformed plasmids into XL1-Blue *E. coli* competent cells (Agilent, https://www.agilent.com). Positive clones were prepared by using DNA-Spin Plasmid Purification Kit (iNtRON Biotechnology) and plasmids. Sanger sequencing used primer walking (Quintarabio, https://www.quintarabio.com). Chromatograms were verified visually to ensure that bases were scored correctly. Full genomes were assembled by using de novo assembly in Geneious version 7.0.6 (https://www.geneious.com).

Because of sample autolysis, full FeLV genomes from 4 panthers (2 contemporary, UCFP228 and UCFP269, and 2 historic, FP115 and FP132) and 1 domestic cat from Florida (x1608) were not recoverable. Partial genome sequencing was performed by using 3 fragments in the *gag* and *env* genes (Table 1). We developed forward and reverse primers to sequence a 115-bp long terminal repeat (LTR)–*gag* fragment, a 98-bp *gag* fragment, and a 121-bp *env* fragment (Table 2).

### FeLV-B Screening Assay

Sequence analysis of FeLV Florida panther UCFP241 full genomes identified FeLV-B. Using FeLV-A, FeLV-B, and enFeLV sequences in GenBank (*30*), we designed a specific FeLV-B PCR and used it to screen all panther samples for FeLV-B (*28*).

### Phylogenetic Analysis

We analyzed full-genome and partial *env* (1–1,294 nt) sequence datasets separately. We compared Florida panther FeLV (FeLV-Pco) and domestic cat FeLV (FeLV-Fca) sequence identity by using the SDTv1.2 nt pairwise comparison tool (*31*). A partial *env* tree was drawn to include as many GenBank FeLV sequences as possible. We aligned the 3 datasets (full genome, concatenated partial genome [3 small segments within the LTR-*gag*, *gag*, and *env* regions from FP115, UCFP228, and UCFP269], and x1608 along with those comparable regions from the FeLV full genomes available, and *env*) by using MUSCLE in MEGA version 5.3 (https://www.megasoftware.net) and manually checked open-reading frames (*32*,*33*).

To investigate phylogeny, we constructed a midpoint-rooted maximum-likelihood nucleotide tree for the full genome and concatenated partial dataset by using 1,000 bootstrap replicates. We determined best-fit substitution models by using jModelTest (*34*) in MEGA version 5.3 and phylogenetic trees constructed in PhyML implemented in SeaView4 (*35*) for the full-genome dataset TN93 + G model (*36*) and the concatenated partial sequencing nucleotide K2 + G model (*37*). A neighbor-joining tree constructed for the *env* dataset by using SeaView4 with a Jukes-Cantor substitution model was rooted with enFeLV and FeLV-B *env* genetic sequences (*38*).

Recombination was not removed from *env* sequences before constructing the tree to clearly demonstrate the phylogenetic relationship of FeLV-B in relation to enFeLV and FeLV-A. For all phylogenetic trees, branches with support <60% were collapsed. Full genomes from UCFP149, UCFP149R1, UCFP231, UCFP275, x1613, and partial sequences (LTR-*gag*, *gag,* and *env*) from FP115, FP132 (3,688 to 8,396 nt), UCFP228, UCFP269, and x1608 are available in GenBank (*30*) (Table 1). Pairwise identity of full genomes were compared for FeLV-Pco, FeLV-Fca, and FeLV-B (GenBank accession nos. JF957361 and JF957363; isolated in the United Kingdom) by using Sequence Demarcation Tool version 1.2 (*31*).

## Results

### FeLV Diagnosis and Case Attributes

We determined prevalence and 95% CIs for FeLV diagnosed in Florida Panthers during 3 periods (Table 3). FeLV was first detected in the Florida panther in 2001 (*19*,*39*). This outbreak affected panthers residing mainly in protected areas in Florida (Figure 1), including Florida Panther National Wildlife Refuge, Big Cypress Swamp, and Okaloacoochee Slough State Forest (*24*). During 2001–2004, ≈131 animals were tested for FeLV, and 5 (3.82%) were found to be FeLV antigen positive (*19*). During 2004–2010, ≈125 animals were negative. The first FeLV case in Florida panthers after the initial outbreak was documented in a road-killed panther in December 2010. During 2014–2016, five additional FeLV-positive free-ranging Florida panthers were identified west of the historic outbreak in more populated areas (Figure 1, panel B). One of these panthers (UCFP231) might have died from infection with FeLV. Cause of death for the other 5 panthers was collision with a vehicle. Contemporary cases identified during 2010–2016 (6 of 184) primarily represented animals hit by vehicles in human populated areas (Figure 1). Prevalence (3.26%) was similar to prevalence during 2001–2004 (Table 3). Lymphadenopathy was documented in 2 of 6 animals (UCFP149, UCFP275). Animal UCFP149 had a linear stomach ulcer. The other animals were too autolyzed to identify gross abnormalities. Confirmatory FeLV PCR and antigen ELISAs reestablished positive and negative diagnoses for the contemporary outbreak.

**Table 3:** Detection of 2 outbreaks of FeLV infection in Florida panthers*

Time frame	No. positive animals/no. tested	Prevalence, % (95% CI)
2001–2004	5/31	3.82 (1.64–8.62)
2004–2010	0/125	0 (0–2.98)
2010–2016	6/184	3.26 (1.50–6.93)

*Values are for historic and contemporary FeLV cases. Animals were tested by using a commercially available test that detects FeLV antigenemia. Animals tested before 2001 were uniformly negative. FeLV, feline leukemia virus.

### Identification of Recombinant Subgroup FeLV-B

A clone sequenced from sample UCFP241 was identified as the recombinant subgroup FeLV-B. An *env* alignment of FeLV-Pco, FeLV-Fca, and enFeLV (Appendix Figure 1) confirmed an infection of FeLV-B resulting from a previously reported recombination event between FeLV-A and enFeLV. Multiple clones were sequenced to confirm presence of FeLV-B. All other Florida panther samples were screened for FeLV-B by PCR (*28*) but were negative.

### Pairwise Comparison and Phylogenetic Analysis

FeLV-Pco showed ≈75% identity at the nucleotide level with published FeLV-Fca and ≈94%–98% identity at the nucleotide level with FeLV isolated from domestic cats in Florida (x1613, x2653, and x2655). The Florida panther FeLV-B full genome sequenced from x2272 showed ≈75% identity with sequenced FeLV-B and 95%–97% identity with FeLV-Pco subgroup A viruses (Appendix Figure 2).

#### Full-Genome Phylogeny

**Figure 3:**
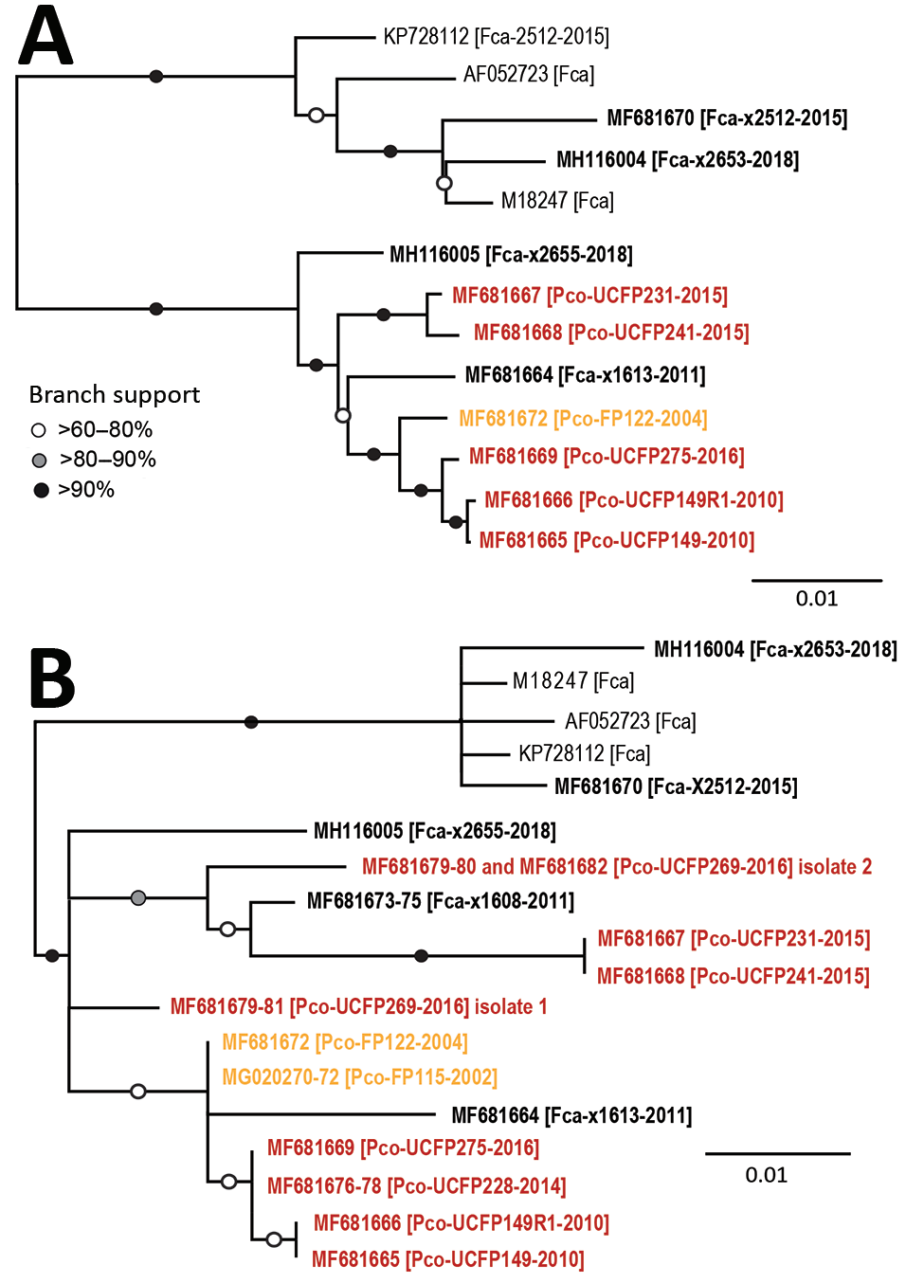
Maximum-likelihood phylogenetic trees showing 2 distinct FeLV-Pco clades in Florida panthers, Florida, USA. A) Full-genome phylogeny indicates Florida FeLV-A sequences are monophyletic. Historic and contemporary FeLV outbreak sequences reside in 1 clade, and a second clade consists solely of contemporary FeLV outbreak sequences. B) Genotyping sequence phylogeny generated from concatenating 3 regions of ≈100 bp (LTR-*gag*, *gag,* and *env*) compares full-genome isolates demonstrated in panel A in addition to 4 individual sequences. Black text indicates FeLV from domestic cats, orange indicates FeLV from panthers during the historic outbreak (2002–2004), and red indicates FeLV from panthers during the contemporary outbreak (2010–2016). Bold indicates isolates sequenced in this study. GenBank accession numbers are provided. FeLV, feline leukemia virus; LTR, long terminal repeat.

The maximum-likelihood tree of full FeLV-Pco and FeLV-Fca genomes documents 2 monophyletic lineages: a lineage composed of FeLV isolates from Florida (FeLV-Fca and FeLV-Pco) and a lineage composed of published FeLV-Fca isolates from other geographically distinct locations in the United Kingdom and the United States. FeLV-Fca isolate (x2655) from Florida groups basal to the 2 FeLV-Pco clades but within the larger Florida FeLV lineage. FeLV-Fca isolate x1613 is basal to the clade that includes all historic and related contemporary isolates. FeLV-Fca isolate x2512 was recovered as part of this study originating from Maryland and groups with FeLV-Fca isolate x2653 as part of a relatively homogeneous United States/United Kingdom clade (Figure 3, panel A).

FeLV-Pco isolates fall into >2 clades. Two isolates from the contemporary outbreak are monophyletic (UCFP231 and UCFP241) and are referred to as contemporary FeLV-Pco. The other FeLV-Pco clade contains 2 FeLV-Pco isolates from the contemporary outbreak (UCFP149 and UCFP275) and is most closely related to the historic FeLV-Pco isolate FP122; this group is referred to here as historic FeLV-Pco.

#### Partial Genotype Phylogeny

Partial genotyping FeLV-Pco sequences documented 10 single-nucleotide polymorphisms (SNPs) and a 9-nt insertion in the untranslated LTR-*gag* region, 3 SNPs in the *gag* region, and 8 SNPs in the *env* region. Phylogenetic analysis of the short concatenated sequence supported similar relationships established by full-genome nucleotide trees (Figure 3, panel B). An additional historic FeLV-Pco (FP115) is most related to other historic FeLV-Pco isolates. FeLV-Pco from a contemporary outbreak sample (UCFP228) also falls in the historic FeLV-Pco clade. Five clones sequenced from the *env* portion of FeLV-Pco UCFP269 showed 2 genotypes. Concatenated sequences from UCFP269 showed a paraphyletic relationship (Figure 3, panel B). Both isolates clustered outside the historic and contemporary FeLV-Pco strain clades (Figure 3, panel B). FeLV-Fca (x1608) groups within the major Florida FeLV clade (Figure 3, panel B).

#### Phylogeny of *env*

**Figure 4:**
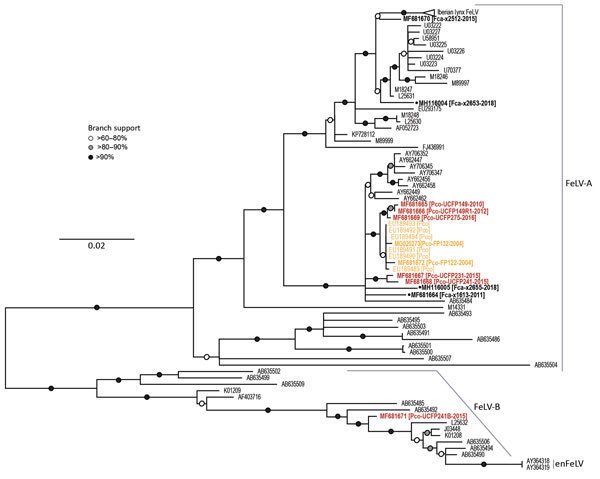
*Env* phylogenies support relationships established in the full-genome tree and document the Florida panther FeLV-B relationship to other known recombinant viruses in Florida panthers, Florida, USA. The *env* tree shows FeLV-A, FeLV-B, and enFeLV sequences (neighbor-joining analysis). One Florida panther sequence (MF681671) can be found in the FeLV-B cluster, identifying it as the recombinant subgroup. Black text indicates FeLV from domestic cats, orange indicates FeLV from panthers during the historic outbreak (2002–2004), and red indicates FeLV from panthers during the contemporary outbreak (2010–2016). Bold indicates isolates sequenced in this study. Black dots indicate sequences from domestic cats in Florida. GenBank accession numbers are provided. en, endogenous; FeLV, feline leukemia virus.

Phylogenetic relationships established by full-genome and concatenated partial sequence trees were supported by the *env* neighbor-joining tree (Figure 4). The *env* phylogeny clearly shows differentiation between FeLV-A/FeLV-B/enFeLV subgroups. FeLV-B and enFeLV subgroups cluster together, and FeLV-A remained a monophyletic group. All FeLV-Pco sequences detected during the historic outbreak are monophyletic (*24*). Two *env* sequences described in this study are closely related to previous sequences reported from the same animal. FP132, an isolate from a panther sample obtained in 2004, has 2 SNPs; FP122, also obtained in 2004, has 5 SNPs. FeLV-Pco sequences clustered into 2 clades, supporting previously identified relationships. One FeLV-Pco clone (UCFP241B) clusters in the FeLV-B clade.

## Discussion

Two distinct FeLV outbreaks were recorded in the Florida panther population during 2001–2016 (Table 3). Phylogenetic data from both outbreak documents that FeLV-Pco resulted from initial spillover from domestic cats, presumably during a predatory event (*24*,*39*). Although FeLV prevalence in domestic cats is relatively low (≈4%) in Florida (*40*), it has been reported that domestic cats are ≈5% of the Florida panther diet (M. Cunningham et al., unpub. data). The relatively high number of domestic cats consumed, particularly in panther habitat proximal to human development, presents opportunities for FeLV spillover from cats to panthers (*40*).

Genomic analysis of contemporary FeLV-Pco identified 3 independent isolates (UCFP149, UCFP149R1, and UCFP275) that were genetically similar to historic FeLV-Pco (FP122; Figure 3, panel A). Analysis also detected FeLV in a paraphyletic clade that is similar to, but distinct from, historic FeLV-Pco (>97% nt identity with FP122 (Appendix Figure 2). This genotype was detected in 2 panthers sampled in 2015 (UCFP231, UCFP241; Figure 3, panel A). A third FeLV strain might be present in contemporary samples and represented by partial FeLV sequences derived from 1 panther (UCFP269; Figure 3, panel B). Three Florida FeLV-Fca isolates (x1613, x2655, and x1608) clustered with Florida panther genotypes. One Florida FeLV-Fca (x2653) isolate was strongly divergent from other Florida FeLV-Fca isolates and resembled previously characterized FeLV-61E and domestic cat FeLV isolates from the United States and the United Kingdom. Movement of domestic cats by owners likely results in mixing of FeLV strains beyond geographic sites.

Full-genome and concatenated partial genome trees demonstrate that domestic cat FeLV strains are situated basal to FeLV-Pco, providing evidence of a domestic cat origin of the panther FeLV infections. Genetic distances between these Florida FeLV-Fca isolates and FeLV-Fca isolates from locations other than Florida indicate a more distant evolutionary relationship between domestic cat strains and Florida FeLV-Fca and FeLV-Pco strains (Figure 3). These findings suggest that minimal species adaptation is required for cross-species transmission between cats and panthers. Additional FeLV full-genome samples would enable a Bayesian ancestral reconstruction analys to further resolve FeLV isolate ancestry.

Full-genome phylogenetic analysis supports the combination and hybrid panther FeLV reemergence hypotheses (Figure 2, panels C, D). Assuming that the Florida panther population was ≈300 animals during 2004–2010, sampling 125 of these animals with no FeLV detected provides a >95% CI that FeLV prevalence was <3%. Test results during both outbreak periods indicated an FeLV prevalence of ≈3%, which indicated that control measures initiated during the historic outbreak were successful in at least controlling, if not eliminating, additional panther FeLV infections for several years. Contemporary FeLV in Florida panthers was identified near human population centers where exposure to feral domestic cats would be more likely to occur (*41*). Although it is feasible that each contemporary case represented an individual exposure to a different domestic cat, panther-to-panther transmission cannot be excluded, particularly for cases that occurred around the same time and showed similar genotypes (i.e., UCFP231 and UCFP241 sampled in 2015; UCFP228 sampled in 2014; and UCFP275 sampled in 2016).

In addition to the common horizontally transmissible FeLV subgroup (FeLV-A), we recovered and sequenced an oncogenic FeLV subgroup (FeLV-B) from tissues from a contemporary Florida panther (UCFP241B; Figure 3, panel B; Appendix Figure 1). This subgroup is a recombinant of FeLV-A and enFeLV, an endogenous retrovirus harbored only by members of the genus *Felis*. Identification of FeLV-B infection in a Florida panther is only possible as a result of horizontal transmission of FeLV from a domestic cat because panthers lack enFeLV to support recombination (*22*). FeLV-B is common in domestic cats; recombination occurs in ≈33%–68% of cats infected with FeLV-A (*42*), presumably by independent recombination events that occur de novo after infection of domestic cats with FeLV-A.

FeLV-B horizontal transmission has been described only on 3 previous occasions (*43*). One study reported that FeLV-B was detected in a jaguar (*Panthera onca*); however, this analysis was based on a sequence amplified from a 232-bp region of the LTR (*12*,*21*). We have clearly documented a full FeLV-B genomic sequence in an endangered non-*Felis* cat species. This finding is of concern because FeLV-B is oncogenic and associated with increased illness and death in domestic cats (*6*,*44**,**45*). Because non-*Felis* spp. Cat species lack enFeLV, they might be more vulnerable to an adapted FeLV-B that is readily horizontally transmitted between animals. Thus, spillover of FeLV-B from domestic cats co-infected with this recombinant strain could represent a greater risk for vulnerable nondomestic cat populations.

Besides individual and population health effects, a potential outcome of FeLV infection in nondomestic felids is germline infection leading to endogenization. Early endogenization results in an infection in which the virus has yet to accumulate mutations rendering the endogenous retrovirus defective; therefore, at this stage, the virus might be passed horizontally to other animals (*46*). Early endogenization might result in decreased fitness, as exemplified by endogenization of koala endogenous retrovirus (*46**,**47*). Infection with this virus has also led to higher incidence of secondary infections, such as chlamydiosis and neoplasias (*48*). Therefore, FeLV infection of panthers and other non-*Felis* cat species is a greater concern for long-term population effects.

Our study demonstrated that even with efforts to control FeLV in an intensively managed population, FeLV remains a risk to Florida panthers, particularly for animals inhabiting areas near urban centers. Moss et al. reported that the proportion of diet consisting of domestic animals is increasing for Colorado pumas, which is concurrent with puma co-localization in human habitats (*49*). This trend is likely present in Florida.

This report highlights the need for continued surveillance of Florida panthers for exposure to FeLV as a major risk management strategy (*40*). Annual sampling of a proportion of the Florida panther population that is sufficient to detect an FeLV incidence of 3% with relative certainty and increased vaccination of panthers and domestic cats along sites of potential interaction are recommended measures to protect against future outbreaks.

Appendix Additional information on multiple introductions of feline leukemia virus in endangered Florida panthers.
