# Correction: Vol. 25, No. 1

**DOI:** 10.3201/eid2502.C22502

**Published:** 2019-02

**Keywords:** errata, erratum, correction

Table 3 misstated the number of animals tested during 2001–2004 and the first author’s biographical sketch was incorrect in Multiple Introductions of Domestic Cat Feline Leukemia Virus in Endangered Florida Panthers (E.S. Chiu et al.). The article has been corrected online (https://wwwnc.cdc.gov/eid/article/25/1/18-1347_article).

