# Supplementary material for: Multiple Introductions of Domestic Cat Feline Leukemia Virus in Endangered Florida Panthers
Source: Emerg Infect Dis. 2019 Jan;25(1):92–101. doi: 10.3201/eid2501.181347 (PMC6302599; doi:10.3201/eid2501.181347)
Supplement: Appendix — Additional information on multiple introductions of feline leukemia virus in endangered Florida panthers. [file 18-1347-Techapp-s1.pdf]

Article DOI: https://doi.org/10.3201/eid2501.181347

Multiple Introductions of Feline Leukemia Virus in Endangered Florida Panthers

Appendix

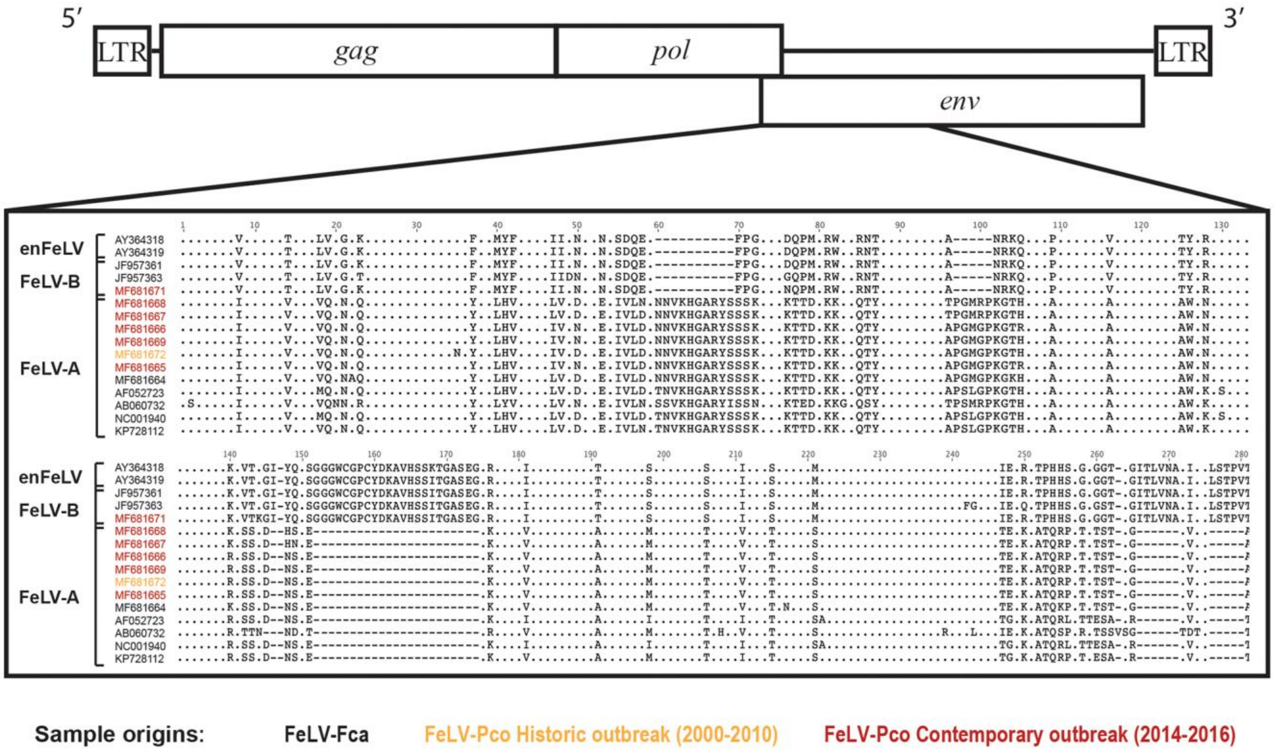

Appendix Figure 1. FeLV Env alignment of FeLV-A, FeLV-B, and enFeLV from published sequences and Florida panthers that support identification of FeLV-B in 1 Florida panther (MF681671). en, endogenous; FeLV, feline leukemia virus; LTR, long terminal repeat.

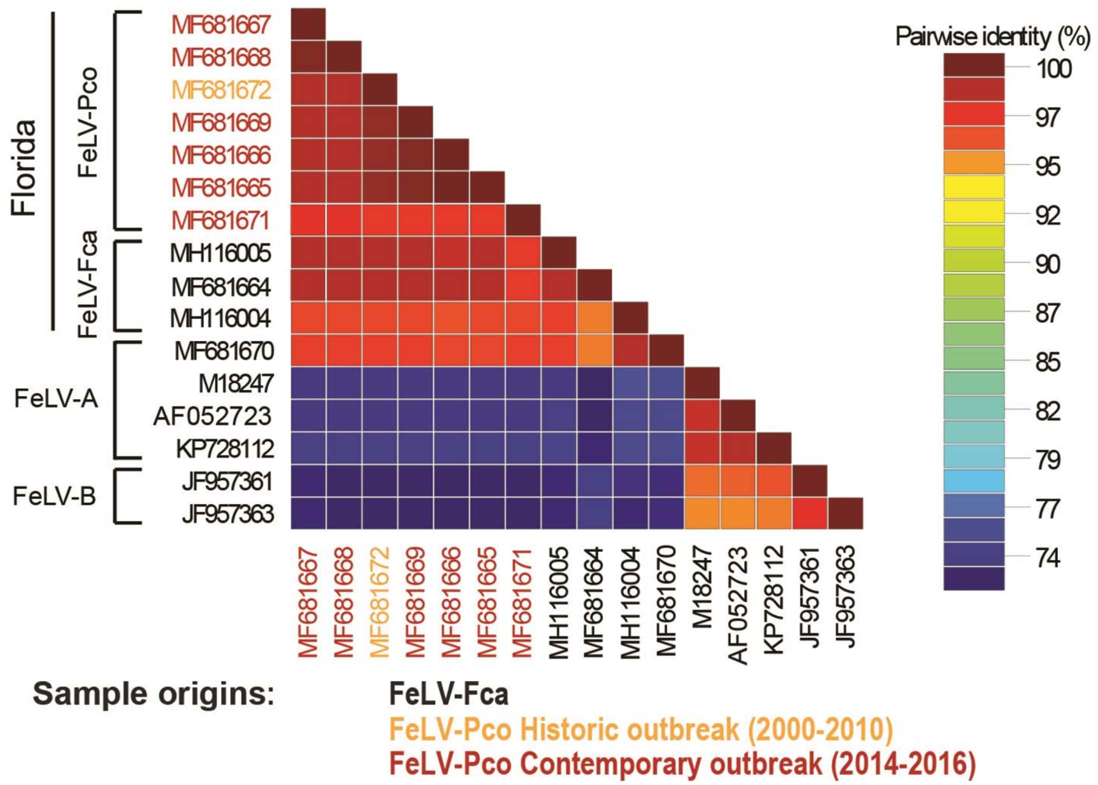

Appendix Figure 2. Heat map of FeLV-Fca and FeLV-Pco showing high homology of FeLV in Florida panthers and domestic cats from Florida. Published FeLV-A show 2 groups of related viruses. FeLV, feline leukemia virus.
